# The Use of Liposomes and Nanoparticles as Drug Delivery Systems to Improve Cancer Treatment in Dogs and Cats

**DOI:** 10.3390/molecules22122167

**Published:** 2017-12-07

**Authors:** Katarzyna Zabielska-Koczywąs, Roman Lechowski

**Affiliations:** Department of Small Animal Diseases with Clinic, Faculty of Veterinary Medicine, Warsaw University of Life Sciences, Nowoursynowska 159c, 02-776 Warsaw, Poland; roman_lechowski@sggw.pl

**Keywords:** cancer, chemotherapy, drug delivery, liposomes, nanoparticles, veterinary medicine

## Abstract

Background: Cancer remains a leading cause of death in companion animals. In human medicine, liposomes and nanoparticles have been extensively investigated as drug delivery systems (DDS) for anticancer agents due to their ability to target cancerous cells and reduce the negative side effects of free cytostatic drugs. In this review, the authors discuss the results of clinical trials using liposomes and polymer-based nanoparticles as DDS to improve cancer treatment in dogs and cats, indicating which ones seem worth further evaluation. The authors then overview ongoing animal cancer clinical trials, evaluating nano-DDS registered on the American Veterinary Medical Association Animal Health Studies Database. Finally, the authors indicate the nano-drugs that require further in vivo evaluation based on the encouraging results obtained from in vitro studies. Conclusions: Liposomes have been the most investigated nano-DDS in veterinary medicine. The lack of cardiotoxicity of the commercially available liposomal doxorubicin (Doxil/Caelyx) suggests it should be used in dogs with cardiac disorders, rather than using free doxorubicin. Cisplatin-incorporated hyaluronic acid nanoparticles, nanocrystals of cisplatin, and paclitaxel are the most promising nano-drugs for potent applications in treating various canine cancers (e.g. oral melanoma, oral sarcoma, and anal gland adenocarcinoma) and their translation into the treatment of human diseases.

## 1. Background

Cancer is the leading cause of death in companion animals, even with the recently registered veterinary anticancer drugs [1]. Fifty percent of dogs above 10 years of age will develop cancer, and 25% of them will die due to neoplasm [2]. In cats, neoplastic disease is less common. However, the treatment of some malignant tumours like feline injection-site sarcomas (FISS), highly invasive neoplasms with local recurrence rates ranging between 14–69%, remains challenging for veterinary practitioners [3]. The method of cancer treatment depends on its histological type and how advanced it is, the availability of each therapeutic method, the general condition of the animal, and the financial status of the owner. In many cases, standard protocols like surgery, radiotherapy, and chemotherapy are ineffective. Chemotherapy may be recommended prior to surgery to decrease tumour size, post-surgery to prevent relapse of the disease, or as a palliative treatment. While it is commonly used in various types of cancer, chemotherapy has many side effects and in many cases is ineffective due to multidrug resistance (MDR). The application of nanotechnology in medicine may solve these problems as nano-carriers can be targeted to specific tissues, reach certain subcellular compartments, or target malignant cells in circulation via active targeting. Nanotherapies can also accumulate or be retained in tumours using enhanced permeation and retention (EPR) or in leaking blood vessels in tumours via passive targeting [4,5,6,7]. Some nanoparticles like gold nanoparticles were shown to avoid P-glycoprotein (P-gp), one of the main efflux pumps responsible for MDR, which can cause chemotherapy failure [8]. Various drug delivery systems (DDS) for anticancer agents such as liposomes, micelles, and albumin-, metal-, and polymer-based nanoparticles were reported to improve the stability of hydrophobic drugs, increase accumulation in cancer tissue, and therefore reduce negative side effects [5,6,7,9,10,11,12,13]. In human medicine, there are few nano-drugs registered in the USA and Europe for cancer treatment, and many others are currently being tested in clinical trials [14].

Nanooncology is a new field in veterinary medicine, with only a few clinical and preclinical studies investigating nano-drugs (Table 1) and their potential application in pet animal cancer treatments, some of which were first performed as translational research for application in human disease. Dogs with spontaneous tumours are good models for human cancer studies, resulting in their use in biodistribution, pharmacokinetic, and efficacy studies on novel anticancer drugs. According to the American Veterinary Medical Association (AVMA) website of animal cancer clinical trials (https://ebusiness.avma.org/aahsd/study_search.aspx), there are currently six clinical trials registered with the use of nanoparticles as DDS to improve anticancer therapy in dogs: nanocrystals of cisplatin (studies No. AAHSD000024, AAHSD000176, AAHSD000370, AAHSD004339), paclitaxel (study No. AAHSD000021), and IL-12 (study No. AAHSD000445) (data collected in August 2017). Each of these studies also has a translational value, as solid tumours arising spontaneously in dogs share many similarities with the same types of neoplasms in humans, for example, canine osteosarcoma (OSA) with paediatric osteosarcomas [15,16,17]. As a result, human cancer patients indirectly benefit from advancing our knowledge of cancer therapy in dogs. Therefore, these studies may be used as proof of concepts and the basis for developing novel anticancer drugs for people.

The main purpose of this review is to summarize the results of clinical trials on the use of nano-carriers to improve anticancer therapy in dogs and cats, indicating the most promising ones. Moreover, the authors present ongoing animal cancer clinical trials on nano-DDS registered in AVMA Animal Health Studies Database and indicate the nano-drugs that need to be further evaluated in vivo based on the encouraging results of in vitro studies using canine and feline cancer cell lines.

### Search Methodology

This review is based on a search of the PubMed database (http://www.ncbi.nlm.nih.gov/pubmed) using the terms “canine” or “feline” and “nanoparticles” or “liposomes” and “cancer”. This review is a synthesis of the current use of liposomes and nanoparticles as DDS in cancer treatments for companion animals and highlights those that warrant further investigation.

## 2. Liposomes

Liposomes are small (sizes vary from 30 nm to several micrometres), spherical, artificial vesicles synthesized from cholesterol and amphiphilic phospholipids, and are the most clinically established nano-DDS [18]. They are biocompatible, biodegradable, and enable the trapping of both hydrophobic and hydrophilic compounds. They increase drug delivery to the tumour site while lowering systemic toxicity [14]. Half of the nano-drugs approved by the FDA for human anticancer treatment are based on liposomal formulations of cytostatic drugs: daunorubicin (Daunoxome) for human immunodeficiency virus (HIV)-associated Kaposi’s sarcoma, doxorubicin (Doxil/Caelyx) monotherapy for HIV-associated Kaposi’s sarcoma and ovarian cancer, as well as in combined therapy with bortezomib for multiple myeloma, cytarabine (Depocyt) for lymphatic meningitis, vincristine (Margibo) for acute lymphoblastic leukaemia, and irinotecan (Onivyde), which was recently approved for metastatic pancreatic cancer [14].

Liposomes are the most commonly investigated nano-carriers both in human and veterinary medicine, likely due to their ease of production and structure modification [19,20,21,22]. Doxil/Caelyx (approved in the USA and Europe, respectively) is around a 100 nm-sized polyethylene glycol-coated (PEGylated) liposomal formulation of doxorubicin, while Myocet, which is around a 180 nm-sized non-PEGylated formulation, is approved only in Europe [23]. Different lipid compositions, sizes, and loading methods of Doxil/Caelyx and Myocet result in varying doses and dose-limiting toxicities, including palmar-plantar erythrodysesthesia (PPES) and myelosuppression, respectively. PEGylation (stealth liposomes) improves the stability and circulation time by increasing water solubility, reducing renal clearance and improving “passive” targeting to tumours. It provides long plasma residence time and decreased reticuloendothelial system (RES) uptake [23]. Until now, of the nanopharmaceutics available on the market, only liposomal doxorubicin (Doxil/Caelyx, Myocet) has been tested in a canine model to assess its pharmacokinetics, biodistribution, and safety [19,20,22,24,25]. A clinical trial of fifty-one dogs with various neoplasms confirmed that Doxil is safe when administered at an intravenous (IV) dose of 1 mg/kg every 3 weeks. Cardiotoxicity, myelosuppression, and gastrointestinal track disorders were not considered dose-limiting toxicities and are normal side effects of treatment with free doxorubicin. The only dose-limiting toxicity observed for Doxil treatment was cutaneous skin reactions, which closely resemble PPES in humans and include: mild erythema, oedema, hyperaemia, alopecia, severe crusting, ulceration, and epidermal necrosis. Fortunately, they were self-limiting and resolved within 1 or 2 weeks [20]. Moreover, Vail and collaborators demonstrated a 25.5% (13 of 51 dogs) overall response to Doxil treatment. Five dogs had complete responses (three dogs with mycosis fungoides, one with oral haemangiosarcoma with lung metastasis, and one with malignant hystiocytosis with regional lymph node metastasis), whereas eight dogs had partial responses (one each with anal sac adenocarcinoma, mammary gland adenocarcinoma, mycosis fungoides, non-Hodgkin’s lymphoma, neurofibrosarcoma, anaplastic sarcoma, fibrosarcoma with lung metastasis, and multiple cutaneous squamous cell carcinoma with lung metastasis) [20]. Results obtained by Vail and collaborators are encouraging. Both should be interpreted with caution, and further studies are needed, as short-term toxicity, not efficacy, was the primary aim of the study. The study also included dogs with various neoplasms, resulting in a small sample size of animals with the same tumour type per group.

On the other hand, the randomized controlled study of thirty-four dogs with splenic haemangiosarcoma did not confirm an increased efficacy of Doxil treatment in comparison to free doxorubicin (each administered 20 mg/m^2^ IV once every 3 weeks for a maximum of 6 treatments). No significant difference in the median survival time (ST) was demonstrated following administration of Doxil and free doxorubicin as an adjuvant therapy following splenectomy [19]. Similar results were obtained by Sorenmo and collaborators, who also showed a lack of improvement in the outcome of fourteen dogs with splenic haemangiosarcoma after intracavitary administration of Doxil [24]. Importantly, both studies demonstrated fewer omental and serosal metastases in dogs treated with Doxil. As a result, researchers claim that patients with tumours that are characterized by an omental or mesenchymal metastasis pattern, such as sarcomatosis or carcinomatosis, may benefit from Doxil treatment. Further studies are necessary to confirm this hypothesis. Nevertheless, both of the studies confirmed that Doxil does not induce cardiotoxicity nor myelosuppression in dogs, and as a result might be considered as an alternative drug to free doxorubicin, especially for dogs with heart disease.

PEGylated liposomal doxorubicin (Caelyx) is proposed as an effective radiosensitizer for palliative treatment of advanced FISS. In a study by Kleiter and collaborators on ten cats, seven (70%) had a partial or complete tumour response, in which radiation was given daily in 5–7 fractions (6 MeV photons, 5–14 MeV electrons) and Caelyx was given once at the dose 1 mg/kg IV prior to the second dose of radiation (in eight cats) or prior to the last three fractions (in two cats) [22].

There is also a single case report demonstrating high efficacy of non-PEGylated liposomal doxorubicin treatment (Myocet) in a dog with chemotherapy-resistant plasma cell myeloma, in which a complete response was demonstrated with the dose of 35 mg/m^2^ administered IV every three to six weeks [25]. A long-lasting response (106 weeks) to non-PEGylated liposomal doxorubicin was assumed to be not only a result of increased local drug delivery to the tumour via passive targeting and increased exposure time to free drug, but also due to avoiding P-gp [25]. Non-PEGylated liposomal doxorubicin was also confirmed to be safe and to decrease the negative side effects (cardiotoxicity, gastrointestinal disorders) of free doxorubicin in clinical trials performed on healthy beagle dogs [26,27,28].

Despite studies on commercially available liposomal doxorubicin, in which low temperature-sensitive liposomes were used as DDS for doxorubicin and administered (0.7–1.0 mg/kg IV 2 or 3 times during a 3-week interval) along with local hyperthermia in dogs with soft tissue sarcomas (*n* = 20), disease stabilization and partial responses were obtained in 60% and 30% of animals, respectively. This result is much better than previous results from Caelyx or Doxil, which warrant further investigation [21]. Nevertheless, the results from low temperature sensitive-liposomes should be interpreted with caution as tumour response was not a main focus of the study, and tumours varied greatly in clinical and histopathological presentations, which may have made the response rate uninterpretable [21]. Moreover, the influence of hyperthermia alone on tumour response was not assessed. However, in opposition to ‘stealth’ liposomes, using low temperature-sensitive liposome doxorubicin (LLD), toxicity included mainly cardiotoxicity, myelosuppression, and liver disorders, making LLD more comparable to free doxorubicin than to ‘stealth’ liposomes [21]. Taking the negative side effects of LLD into consideration, for dogs with cardiac or liver disorders, ‘stealth’ liposomes seem to be a better DDS due to their lower toxicity than doxorubicin.

There is also a one randomized study of forty dogs with spontaneous OSA treated with cisplatin encapsulated in ‘stealth’ liposomes (SPI-77) (350 mg/m^2^ or 300 mg/m^2^ IV every 3-weeks for four treatments) as an adjuvant therapy of amputation; however, it failed to prove its effectiveness. No significant difference in disease-free survival (DFS) or overall survival (OS) was seen between dogs that received SPI-77 and those that received carboplatin instead of the test compound, or between dogs treated with SPI-77 and the prospective group treated with cisplatin alone [29].

Recently, when beagle dogs were used to assess the decreased pharmacokinetics of liposome encapsulated vincristine (L-VCR), they showed an increased therapeutic index and higher concentration of L-VCR in comparison to free vincristine after single IV injection [30]. Liposomes were also suggested to be an efficient DDS for lung delivery. In a study performed on 25 healthy dogs receiving paclitaxel liposomes (negatively charged, 501.60 +/− 15.43 nm in diameter), a higher accumulation in the lungs was determined in comparison to free paclitaxel [31].

Non-protein lipid nanoemulsion is similar to low protein lipoprotein, receptors for which are overexpressed in cancer cells [32]. Lucas and collaborators claimed that it is a safe DDS for carmustine when it is in combination with vincristine and prednisone for treatment of canine multicentric lymphoma [33]. In a pilot study of fifteen dogs, the effectiveness of this carmustine nanoemulsion was similar to free drug. With good tolerability and minimal side effects of this novel drug, it should be further evaluated for efficacy [33].

In summary, further clinical trials on liposomes as DDS should be performed on large numbers of animals with the same tumours type. In the case of solid tumours, drug response should be evaluated according to the RECIST criteria [1].

## 3. Liposomes for Immunotherapy and Gene Delivery

Despite liposomes being used as DDS for cytostatic drugs, they are also used as a smart nano-vehicle for immunotherapy and gene delivery. Clodronate is a first-generation bisphosphonate that is used in the clinic for prevention of the development of bone metastases or excessive bone resorption, as well as for the treatment of inflammatory diseases such as osteoarthritis. Nevertheless, recently it has been proven that clodronate encapsulated in liposomes has the ability to supress tumour growth and metastasis by depletion of tumour-associated macrophages (TAM) [34]. Hafeman et al. showed, in both in vitro and in vivo studies, that liposomal clodronate (LC) is an effective agent against canine haemangiosarcoma, as it depletes macrophages and has the ability to kill cancerous cells through apoptosis [35]. Dogs (*n* = 5) with spontaneous haemangiosarcomas that previously failed conventional chemotherapy with prednisolone and lomustine were enrolled in a study using 0.5 mL/kg LC, which was administered by IV every other week. There were no systemic, adverse side effects after LC therapy (except for one dog that presented with a temporary, short-lasting fever), and two of five dogs had significantly reduced tumour size. Moreover, the LC killing ability was demonstrated to be closely related to the uptake of liposomes, which indicates that liposomes may be considered a good DDS for histiocytic neoplasms [35]. However, as only 5 dogs were included into the study, a multi-centre clinical trial on a larger number of animals should also be performed.

Muramide-Tripeptide-phosphatidylethanolamine (MTP-PE) is an immunomodulator that activates monocytes and macrophages to become cytotoxic to cancerous cells. MTP-PE encapsulation into liposomes enhances the endocytosis and tissue uptake by the mononuclear phagocyte system and prolongs its half-life in circulation [36]. The first positive results of using Liposomal Muramide-Tripeptide-phosphatidylethanolamine (L-MTP-PE) for prevention of distant metastasis of canine osteosarcoma were reported on fourteen dogs who received L-MTP-PE after amputation of the affected limb. MacEwen and collaborators demonstrated that L-MTP/PE (2 mg/m^2^ IV, administered twice a week for 8 weeks) significantly prolongs ST and the metastasis free interval (MFI) (median MFI was 168 and 58 days and median ST was 222 and 77 days for L-MTP-PE and free liposomes, respectively) in the treatment group compared to the control group (thirteen dogs that received empty liposomes after surgery) [37]. Kurzman et al. obtained similar results when using the same therapeutic schedule of L-MTP-PE administration on twenty-five dogs with OSA previously treated with cisplatin chemotherapy (70 mg/m^2^ IV every 28 days, 4 times) [38]. Both ST and MFI were prolonged for dogs treated with L-MTP-PE after surgical amputation compared to dogs who underwent surgery alone and had no evidence of metastasis at the time of enrolment (from 9.8 months to 14.4 months and 7.6 months to 11.2 months for median ST and median MFI, respectively). On the other hand, when L-MTP-PE (2 mg/kg IV, once or twice weekly) was administered concurrently with cisplatin chemotherapy (70 mg/m^2^ IV every 21 days for 4 doses) there were no significant differences in median survival time [38]. Although the contradictory results of immunotherapy with L-MTP-PE combined with standard chemotherapy are also described for treatment of children diagnosed with OSA, L-MTP-PE is approved in Europe to use together with chemotherapy for treatment of newly diagnosed OSA in children, as some patients may benefit from it [39,40,41].

On the other hand, the multi-center clinical trial of thirty-two dogs with splenic haemangiosarcoma showed that dogs that received 4 cycles of L-MTP-PE (first dose 1 mg/m^2^ , then 2 mg/m^2^ IV) together with doxorubicin (30 mg/m^2^, IV) and cyclophosphamide (100 mg/m^2^ IV) once every 3 weeks, starting from 2 weeks following splenectomy, had a significantly prolonged DFS (median DFS were: 188 and 127 days for L-MTP-PE and free liposomes, respectively) and OS (median OS were: 277 and 143 days for L-MTP-PE and free liposomes, respectively) compared to dogs that received free liposomes instead of L-MTP-PE [36]. The treatment was more effective for dogs with clinical stage I disease compared to dogs with clinical stage II [36]. Similarly, in randomized clinical trials testing oral melanomas in dogs, L-MTP-PE showed promising anti-metastatic activity only in early stages of the disease (ST increased for more than 2 years), in comparison to in advanced-stage canine oral melanoma, in which L-MTP-PE was administered alone post-surgery or together with recombinant canine granulocyte macrophage colony-stimulating factor, neither resulting in a significant antitumour response [42]. Furthermore, MacEwen and collaborators claimed that increasing the frequency of L-MTP-PE administration (from once to twice a week) did not influence the therapeutic response [42]. Results obtained suggest that the success of additional therapies with L-MTP-PE is probably associated with the staging of the disease, with much better responses in the early stages of cancer.

There is also one study of cats with mammary gland tumours in which L-MLT-PE was tested as a non-specific immunomodulator for application in treating female breast cancer. When L-MTP-PE (2 mg/kg IV, once a week for 8 weeks) was given to forty cats that underwent total mastectomy, results showed that cats with stage II disease had a statistically significant longer DFI (*p* < 0.02) and OS (*p* < 0.005) when compared to cats with stage III mammary gland tumours [43]. However, in general L-MTP-PE treatment after radical surgery of mammary adenocarcinomas in cats had no influence on DFI or OS. Why the feline and not canine model of mammary cancer was chosen to perform this study remains questionable. Due to homology between the canine and human genome sequences, as well as similarities in morphology, biological behavior, and molecular biology between human and canine mammary gland tumours, the canine, not feline, model has been accepted as an excellent model for woman breast cancer [44,45,46].

The review presented results on both canine and feline immunotherapy with L-MTP-PE and highlights how important is to perform clinical staging of the disease before making critical treatment decisions. Although nanomaterials as DDS may improve drug delivery to solid tumours, the early detection of cancer is a key factor for achieving a good therapeutic response.

Recently, in human medicine, IL-2 has been shown to be the first effective immunotherapeutic providing long-lasting, complete regression for patients with metastatic melanoma and renal cancer [47]. It is also an effective treatment for various animal tumours. However, its main limitation is a narrow therapeutic index, especially after an IV bolus administration. As a result, the possibility of encapsulating IL-2 in liposomes to reduce its negative side effects is currently being investigated. In veterinary medicine, in a pilot study of five dogs, IL-2 liposomes were shown to be well tolerated (in all dogs) and efficacious (two of five dogs had a total response) using the inhalation therapy (twice daily for 30 days) for pulmonary metastases of OSA [48]. Lung metastasis is the main cause of death in dogs with OSA. Khanna and collaborators established, in both in vitro and in vivo studies, that during the use of inhalation therapy, IL-2 liposomes stimulate pulmonary immune antitumour activity much more than free IL-2 [49]. Although, due to a very small number of animals included in the study, the final conclusion of its efficacy cannot be made, according to its ease of implementation and promising results, IL-2 should be further tested.

Another novel therapeutic approach involves the use of liposomes for IV gene delivery. IV gene delivery using liposome-DNA complexes (LDC) was shown to elicit non-specific antitumour activity and inhibit tumour angiogenesis in dogs with soft tissue sarcomas and OSA lung metastasis [50,51]. A study of twenty dogs with metastatic OSA indicated that canine IL-2 cDNA encapsulated into liposomes is safe and well tolerated in low doses (20 µg/kg of plasmid DNA) and prolongs OS in comparison to untreated dogs [50]. Results from a study on twelve dogs with soft tissue sarcomas suggest that empty LDC themselves present antitumour activity mediated by triggering an immune response that resulted in tumour suppression [51].

## 4. Polymer-Based Nanoparticles

Besides liposomes, the biocompatible polysaccharide hyaluronan (HA), which is approved by the FDA for use in humans, is of interest as a safe, novel, less toxic, and efficacious alternative DDS for cisplatin treatment of various canine tumours. HA is a ligand for the CD44 receptor and is cleared by the lymphatic system through endocytosis followed by lysosomal degradation. As many cancer cells overexpress the hyaluronan receptor CD44, intratumoral injection of cisplatin polysaccharide hyaluronan (HA–Pt) results in a greater uptake of the nano-drug by those cells [52]. A pilot study on the pharmacokinetics of the cisplatin hyaluronan nanoconjugate (HA–Pt) (1.5 mL of nanocarrier and 20 mg of cisplatin) after a single intratumoral injection showed a 1000-fold and 100-fold higher HA–Pt concentration in treated tumours and local lymph nodes, respectively, than in plasma [53]. Both canine and murine models lacked systemic toxicity of HA–Pt [52,53]. Furthermore, in a phase I clinical trial of sixteen dogs with spontaneous tumours, three dogs reached complete response (two had oral and one nasal planum squamous cell carcinoma) and three dogs had stable disease after intratumoral or peritumoral HA–Pt injections (10–30 mg/m^2^, 1–4 intratumoral or peritumoral injections at 3 week-intervals), which is better than previously reported studies of free cisplatin. Although the toxicity rate for this study was high (60%), the authors explained it as a result of poor compound purification prior to performing the trial, resulting in diaquated impurities [54]. Interestingly, no nephrotoxicity, the main adverse effect of cisplatin treatment, was noticed in any of the dogs tested. This is opposed to liver damage, an extremely rare side effect of standard cisplatin treatment, which resulted following HA–Pt administration. Hepatotoxicity probably appeared due to the large size of polymer-based HA–Pt, which could not be cleared through the kidney, and the HA, which is normally metabolized in the liver, that was used as the DDS [54]. Based on these primary results, a clinical trial on intratumoral injections of HA–Pt was recently approved for the treatment of mouth cancers (melanoma and sarcoma) in dogs (study No. AAHSD000024), and in combination with palliative radiotherapy for the treatment of various non-resectable canine solid tumours (studies No. AAHSD004339 and AAHSD000370). They focus on the tolerability, safety, and efficacy that will be measured according to the RECIST criteria. Potential benefits of intratumoral injections such as higher local drug retention and decreased systemic toxicity compared with systemic routes are mentioned by investigators. The limitations of intratumoral administration of test compounds especially in case of very aggressive, metastatic disease should be taken into consideration. Recently, Feldhaeusser and collaborators reported the possiblilty of using biocompatible polymeric nanoparticles based on biodegradable poly(lactic-co-glycolic acid) (PLGA)-block(b)-PEG functionalized with a terminal triphenyl-phosphonium cation to target the mitochondria of hyperpolarized cancer cells as a DDS for Platin (M), a prodrug of cisplatin that allows mitochondrial targeting of hyperpolarized cancer cells for the treatment of brain tumours, thereby reducing side effects while enhancing efficacy [55,56,57]. In vitro studies demonstrated their enhanced cytotoxic effect on canine glioma and glioblastoma cell lines in comparison to free cisplatin. Additionally, in vivo studies in murine models demonstrated that these drug-nanocarriers are capable of crossing the blood-brain barrier, which is one of the limitations of using of standard cisplatin therapy. Toxicity studies on six healthy beagle dogs after single IV administration (0.5 mg/kg) verified it is non-toxic, which encourages further studies to assess its therapeutic potential for dogs with brain tumours [57].

## 5. Nanocrystals

Nanocrystals of two anticancer drugs, cisplatin and paclitaxel, are currently being investigated according to the AVMA animal cancer clinical trials website to test their possible application in veterinary medicine. The proof of concept study (No. AAHSD000176) on the use of the nanocrystal of cisplatin for apocrine gland anal sac adenocarcinoma (AGASACA) in dogs is being performed to demonstrate the feasibility of its localized subcutaneous administration and to demonstrate that this nano-drug accumulates in tumour tissue and local lymph nodes. Nanocrystal paclitaxel (CTI-52010, Crititax, consisting of drug and saline) will be evaluated in dogs with various tumour types, excluding OSA, for the proper doses and its efficacy after subcutaneous injections. CTI-52010 will be examined for the prevention of allergic reactions that appeared during standard paclitaxel administration and for possible reduction of tumour size. In the preliminary studies on three healthy dogs, Axiak and collaborators determined its maximal tolerated dose (120 mg/m^2^) with neutropenia as a dose-limiting toxicity [58]. Previously performed in vitro studies on canine and human prostate cancer cell lines (Ace-1 and PC3, respectively) demonstrated the ability of CTI-52010 to decrease cell viability and cell survival, induce apoptosis, and cause the rigidity of microtubules in both human and canine castration-resistant prostate cancer [59].

## 6. Gold Nanoparticles

Gold nanoparticles (AuNPs) have been extensively investigated as DDS because of their physio-chemical, optical, and thermal properties. Even though none have yet to receive FDA approval, they are undergoing testing in several clinical trials in both human and veterinary medicine [14]. Veterinary clinical trials to evaluate the therapy of AurolaseÂ^®^ (gold nanoparticle infusions plus targeted laser treatment) as a smart therapeutic medical device that can selectively destroy solid tumours using near infrared laser illumination have been currently ongoing. However, the main limitation of the use of infrared light in tumour ablation is its limited optical penetration depth within the tumour, depending on the type of tissue, its vascularization and wavelength used [60]. Furthermore, there are various preclinical studies on the use of AuNPs as valuable platforms for drug delivery, with several showing enhanced selectivity and improved efficacy [11,61,62,63]. Recently, the first promising results in veterinary medicine have been shown for doxorubicin conjugation to glutathione-stabilized gold nanoparticles (Au-GSH-Dox) (4.1 nm ± 0.2 nm in diameter) for FISS and Co (II) and Zn (II) compounds: [Zn(1,10-phenanthroline-5,6-dione)_2_]Cl_2_ (TS262) and [CoCl(H_2_O)(1,10-phenanthroline-5,6-dione)_2_][BF_4_] (TS265) conjugated to AuNPs (14 nm in diameter) and functionalized with PEG and bovine serum albumin (BSA) for canine mammary tumours [64,65,66]. They showed enhanced cytotoxicity while lowering the dose of the conjugated drug. However, as 80% of the promising in vitro studies failed during clinical trials, in vivo studies are essential to prove the efficacy and safety of those novel compounds [67].

## 7. Conclusions

Nanooncology is a developing field in veterinary medicine, with only a few clinical and preclinical trials performed in dogs and a single clinical trial performed in cats. Liposomal doxorubicin is the most investigated nano-drug in veterinary medicine, and canine studies were performed prior to its registration. Doxil/Caelyx has shown a longer blood circulation and fewer side effects (mainly a lack of cardiotoxicity) in comparison to free doxorubicin. This is an important finding as cardiotoxicity is the main adverse effect limiting the use of doxorubicin in dogs. This is opposed to its effects in cats, in which no heart toxicity is observed after doxorubicin treatment. Myocet, on the other hand, has similar side effects to free doxorubicin, despite a significant reduction of myelosuppression. Further studies on liposomal doxorubicin should be performed in a large number of dogs with the same tumour type to clearly evaluate its increased efficacy. Liposomes encapsulated with an immunotherapeutic (LMP-PE) showed promising primary results, prolonging OS and DFI in dogs with haemangiosarcoma and OSA. Until now, hyaluronan cisplatin nanoparticles, nanocrystals of cisplatin and paclitaxel that have been recently investigated in U.S. phase I and II animal clinical trials, seem to be the most promising nano-drugs for potent application in the treatment of various canine cancers (e.g., oral melanoma, oral sarcoma, and anal gland adenocarcinoma) and its translation into the treatment human diseases.

## Figures and Tables

**Table 1:** Preclinical and clinical trials on the use of liposomes and nanoparticles as drug delivery systems (DDS) in dogs and cats.

DDS	Active Substance/Market Name	Species	Type of Study	Results	Reference
‘Stealth’ PEGylated liposomes	doxorubicin/Doxil	dog	toxicity and efficacy	maximal tolerated dose: 1 mg/kg IV every 3 weeks; the dose-limiting toxicity: cutaneous toxicity resembling palmar-plantar erythrodysesthesia, lack of significant neutropenia or cardiomyopathy; an overall response rate was 25.5% (5 of 51 dogs had complete responses and 8 of 51 dogs had partial responses)	[20]
‘Stealth’ PEGylated liposomes	doxorubicin/Doxil	dog	randomized, efficacy and toxicity	no differences in survival time between dogs with splenic haemangiosarcoma after splenectomy treated with Doxil and free Dox as an adjuvant monotherapy; adverse side effects: a desquamating dermatitis like palmar-plantar erythrodysesthesia, anaphylactic reaction; lack of cardiotoxicity	[19]
‘Stealth’ PEGylated liposomes	doxorubicin/Doxil	dog	prospective, unmasked, uncontrolled toxicity, pharmacokinetic, efficacy	intraperitoneal administration of Doxil does not prevent intraabdominal recurrence of haemangiosarcoma in dogs; effective drug concentration is obtained after intraperitoneal administration and its clearance is comparable with IV administration	[24]
‘Stealth’ PEGylated liposomes	doxorubicin/Caelyx	cat	efficacy	response rate: 70%; 2 of 10 cats had complete responses and 5 of 10 cats had partial responses when Caelyx was administered together with daily radiotherapy	[22]
Non-PEGylated liposomes	doxorubicin/Myocet	dog	efficacy-case report	complete response with Myocet (35 mg/m^2^ IV every 3–6 weeks administered 6 times) in a dog with an immunoglobulin A-secreting chemotherapy-resistant myeloma	[25]
Non-PEGylated liposomes	doxorubicin/Myocet	dog	toxicity	no cardiomyopathy	[26]
Non-PEGylated liposomes	doxorubicin/Myocet	dog	preclinical toxicology	dose-limiting toxicity after intraperitoneal administration: chemical peritonitis; other adverse side effects: abdominal toxicity, myelosuppression and thoracic toxicity	[27]
Non-PEGylated liposomes	doxorubicin/Myocet	dog	preclinical toxicology	maximal tolerated dose: 2.25 mg/kg, adverse effect: pyrexia	[28]
Low temperature sensitive liposomes (LTSL)	doxorubicin	dog	toxicity and pharmacokinetic	dose-limiting toxicities: grade 4 neutropenia, acute death secondary to liver failure; adverse side effects: myelosuppression, cardiac failure; maximal tolerated dose: 0.93 mg/kg; response rate: 90% (6 of 20 dogs had partial response and 12 of 20 dogs had stable disease after at least 2 doses of LTSL-doxorubicin (0.7–1.0 mg/kg IV over 30 min) concurrently with local hyperthermia	[21]
‘Stealth’ PEGylated liposomes	cisplatin/SPI-77	dog	randomized, multi-centre efficacy	no differences in survival time in 40 dogs with spontaneous osteosarcoma that underwent limb amputation after adjuvant SPI-77 administration compared to carboplatin therapy	[29]
Liposomes	vincristine	dog	pharmacokinetic	increase therapeutic index of liposomal vincristine after single IV injection 0.07 mg/kg	[30]
Liposomes	paclitaxel	dog	pharmacokinetic and biodistribution	15-fold higher paclitaxel concentration in the lung at 2 h after paclitaxel liposomes IV administration than after free paclitaxel injection	[31]
Polysaccharide hyaluronan	cisplatin	dog	pharmacokinetic	1000-fold greater drug concentration in in tumours than in plasma after intratumoral injection (20 mg of cisplatin in the hyaluronan-cisplatin conjugate)	[53]
Polysaccharide hyaluronan	cisplatin	dog	efficacy and pharmacokinetics	3 of 7 dogs with oral and nasal squamous cell carcinoma had complete response and 3 of 7 dogs had stable disease (dose 10–30 mg/m^2^ intratumoral or into peritumoral submucosa once every 3 weeks, approximately 4 times); adverse side effects: myelosuppression, cardiotoxicity, hepatic toxicosis; lack of nephrotoxicity	[54]
PLGA-block(b)-PEG functionalized with a terminal triphenyl-phosphonium cation	platin (M) ^a^	dog	safety and biodistribution	cross the blood brain barrier and accumulate in the brain; minimal adverse reactions after single IV injection at doses: 0.5 mg/kg, 2.9 mg/kg and 2.2 mg/kg	[57]
Lipid nanoemulsion	carmustine	dog	safety and efficacy pilot study	no difference between the treatment of LDE carmustine and free carmustine; adverse side effect: neutropenia	[33]
Nanocrystal	cisplatin	dog	biodistribution, proof of concept, safety	no results reported: study within recruitment or currently ongoing	[68] ^b^
Nanocrystal	paclitaxel/Crititax	dog	safety and pharmacokinetics	maximal tolerated dose: 120 mg/m^2^, dose-limiting toxicity: 4 grade neutropenia; starting dose for phase I/II clinical trials: 80 mg/m^2^ IV	[58,68^c^]
Glutathione stabilized gold nanoparticles	doxorubicin	cat	in vitro and in ovo efficacy	higher cytotoxic effect of Dox conjugated to glutathione stabilized gold nanoparticles (Au-GSH-Dox) than free Dox in fibrosarcoma cell lines with high activity of P glycoprotein (FFS1WAW, FFS1 and FFS3), significantly reduced tumour size after single intratumoral injection of Au-GSH-Dox	[64,65]
PEG and BSA functionalized gold nanoparticles	Zn(DION_2_)Cl (TS262), CoCl_2_(H_2_O))(DION)_2_[(BF_4_)] (TS265)	dog	in vitro efficacy	higher cytotoxic effect of tested compounds in canine mammary tumour cell line (FR37-CMT) than free Dox or cisplatin	[66]

^a^ Prodrug of cisplatin; ^b^ Study No. AAHSD000024, AAHSD004339, AAHSD000176, AAHSD000370; ^c^ Study No. AAHSD000021.

## Abbreviations

Au-GSH-Dox	doxorubicin conjugated to glutathione-stabilized gold nanoparticles
Au-NPs AVMA	gold nanoparticles American Veterinary Medical Association
BSA	bovine serum albumin
DDS	drug delivery system
DFS	disease free survival
FDA	Food and Drug Administration
FISS	feline injection-site sarcoma
HA	polysaccharide hyaluronan
HA-Pt	cisplatin polysaccharide hyaluronan
IL-2	interleukin 2
IL-12	interleukin 12
IV	intravenous
LC	liposomal clodronate
LDC	liposome-DNA complexes
LLD	low sensitive liposome doxorubicin
L-MTP-PE	Liposomal Muramide-Tripeptide-phosphatidylethanolamine
L-VCR	liposome encapsulated vincristine
MDR	multi drug resistance
MFI	metastasis free interval
OS	overall survival
OSA	osteosarcoma
PEG	polyethylene glycol
P-gp	P-glycoprotein
PLGA	poly (lactic-co-glycolic acid)
PPPS	palmar-plantar erythrodysesthesia
RES	reticulo-endothelial system
ST	survival time
TAM	tumour associated macrophages
